# Effects of Flavonoids on Cancer, Cardiovascular and Neurodegenerative Diseases: Role of NF-κB Signaling Pathway

**DOI:** 10.3390/ijms24119236

**Published:** 2023-05-25

**Authors:** Maria Magdalena Barreca, Riccardo Alessandro, Chiara Corrado

**Affiliations:** Department of Biomedicine, Neuroscience and Advanced Diagnostics (Bi.N.D.), Section of Biology and Genetics, University of Palermo, 90133 Palermo, Italy; mariamagdalena.barreca@unipa.it (M.M.B.); riccardo.alessandro@unipa.it (R.A.)

**Keywords:** flavonoids, NF-κB (Nuclear Factor-κB), cancer, neurodegenerative diseases, cardiac diseases

## Abstract

Flavonoids are polyphenolic phytochemical compounds found in many plants, fruits, vegetables, and leaves. They have a multitude of medicinal applications due to their anti-inflammatory, antioxidative, antiviral, and anticarcinogenic properties. Furthermore, they also have neuroprotective and cardioprotective effects. Their biological properties depend on the chemical structure of flavonoids, their mechanism of action, and their bioavailability. The beneficial effects of flavonoids have been proven for a variety of diseases. In the last few years, it is demonstrated that the effects of flavonoids are mediated by inhibiting the NF-κB (Nuclear Factor-κB) pathway. In this review, we have summarized the effects of some flavonoids on the most common diseases, such as cancer, cardiovascular, and human neurodegenerative diseases. Here, we collected all recent studies describing the protective and prevention role of flavonoids derived from plants by specifically focusing their action on the NF-κB signaling pathway.

## 1. Introduction

Flavonoids are a class of natural compounds characterized by a polyphenolic group and contained in different plants, fruits, grains, and flowers, as well as in beverages such as tea or wine [1]. A healthy diet is recognized all over the world as fundamental to preventing or ameliorating several diseases, such as cardiovascular and neurodegenerative diseases or cancer and its progression [2,3,4]. Hence, they are recognized as dietary flavonoids.

**Figure 1:**
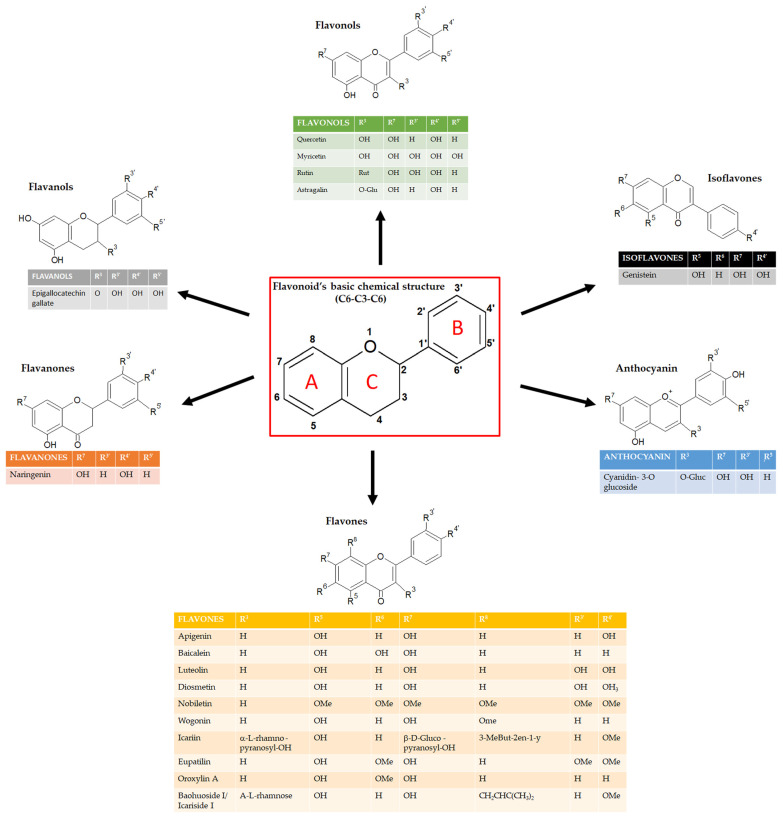
Schematic representation of the chemical structure of flavonoids and its subgroups. The general structure of flavonoids consists of two benzene rings (A and B) linked via a heterocyclic pyrane ring (C).

Flavonoids can be classified into several subgroups depending on the carbon of the C ring on which the B ring is attached and the degree of unsaturation and oxidation of the C ring [5]. According to these differences, we can distinguish isoflavones, neoflavonoids, flavones, flavonols, flavanones, flavanonols, flavanols or catechins, anthocyanins, and chalcones (Figure 1). Some natural compounds contain specific subgroups, e.g., onions and tea are major dietary sources of flavonols and flavones; isoflavones are mainly found in leguminous species; proanthocyanidins or condensed tannins are found in flowers, seeds, and fruits of diverse plant species, such as grapes, apples, blueberry, and some cereals; flavanols (epicatechin), flavonols (e.g., myricetin and quercetin) and anthocyanins (e.g., malvidin-3-glucoside) are enriched in red wine together with the resveratrol, well known for its antioxidant properties [6,7]. For the specific concentration of each flavonoid in the most common foods, refer to the USDA (United States Department of Agriculture) database for Flavonoid Content of Selected Foods (https://www.ars.usda.gov/ARSUserFiles/80400535/Data/Flav/Flav3.3.pdf, accessed on 17 May 2023).

In the last decade, the scientific community’s interest in these compounds has increased, and their beneficial or protective effects against different human diseases have been widely recognized.

Nutraceutical compounds have multiple biological activities, such as antioxidant, anti-inflammatory, or antiplatelet properties. The plant-derived flavonoids have a wide range of activities that could make them particularly effective against cardiovascular and neurodegenerative diseases [2,3]. Moreover, many scientists focused on the anticancer activities of several flavonoids and demonstrated their beneficial effects [8,9,10].

For these properties and together with absent or very low toxicity, they can be a valuable resource for the prevention of several diseases or to reduce tumor progression.

Nowadays, flavonoids are essential components of nutraceutical, pharmaceutical, and cosmetic applications because they have a huge spectrum of biological activity. However, the main mechanism through which they exert these effects is not well known. Recent manuscripts describe the role of flavonoids as anti-inflammatory agents targeting the NF-κB pathway in specific diseases such as cardiovascular diseases or cancer [11,12,13]. The Global Burden of Disease summarizes the main causes of death worldwide, the mortality across countries for a single disease, and the possible correlations with age and sex (https://www.thelancet.com/gbd#healthSummaries, accessed on 17 May 2023). Except for underdeveloped countries, these data, together with that by the global health observatory of the World Health Organization, showed that cardiovascular disease (CVD) is the top cause of death globally, especially in most industrialized countries such as North America and Europe. CVD refers to a plethora of pathologies that include hypertension, coronary heart disease (heart attack), and cerebrovascular disease (stroke), as discussed below. Cancers are nowadays defined as the second cause of death. Cancer is characterized by the fact the cells grow and spread in the body very fast through the blood and lymph systems, becoming a very aggressive and uncontrollable pathology [14]. The Global Burden of Disease recognizes dementia as the third cause of death worldwide. Dementia is a complex disease that includes several forms, such as Alzheimer’s disease, that result in the loss of cognitive capacity, including memory, language, and learning (https://www.who.int/data/gho/data/themes/mortality-and-global-health-estimates, accessed on 17 May 2023).

This review intends to summarize and discuss the results published in the last five years regarding the role of flavonoids on the diseases that affect most people around the world nowadays: cancer, cardiovascular, and neurodegenerative diseases. Moreover, we decided to focus only on the flavonoids that exert their protective or preventive role by affecting the NF-κB (Nuclear Factor-κB) signaling pathway.

## 2. The NF-κB Pathway

The NF-κB proteins can regulate the expression of hundreds of genes, which affect important physiological processes such as inflammation, immunity, proliferation, and cell death [15]. Since NF-κB activity is spontaneously regulated by several different stimuli, NF-κB proteins can be considered important regulators of cellular homeostasis [16,17].

NF-κB is a key mediator in the inflammatory response; its activation promotes the transcription of several target genes, many of which are pro-inflammatory [18]. Two pathways lead to the activation of the NF-κB signaling: the canonical and the non-canonical pathways [18,19]. In both pathways, the activation of the enzyme IκB Kinase (IKK) represents the common regulatory step. Notably, the canonical pathway is the most studied and plays a critical role in inflammatory responses. In this pathway, the NF-κB (p65/p50) dimer, in its inactive state, is sequestered in the cytoplasm by IκB (inhibitor of NF-κB) that covers the nuclear localization signal (NLS) of NF- κB protein.

Upon exposure to pro-inflammatory stimuli, such as cytokines, pathogens, and danger-associated molecular patterns, the p65/p50 dimer is released from IκB due to the phosphorylation cascade resulting in the proteasomal degradation of IκB. Subsequently, p65/p50 translocates into the nucleus, where it promotes the activation and expression of NF-κB target genes [18]. On the other hand, the non-canonical pathway is activated through a subset of Tumor Necrosis Factor Receptor (TNFR) superfamily members, leading to the activation of NF-κB-inducing kinase (NIK). NIK phosphorylates IKKa, which phosphorylates the C-terminal of NF-κB, allowing it to translocate into the nucleus. Once into the nucleus, NF-κB induces the expression of its target genes that play a role in cell proliferation and survival, as well as in immune cells’ development and inflammation, finally inducing the progression of several diseases, as discussed below [18].

## 3. Flavonoids and Cancer: Role of NF-κB Signaling Pathway

Cancer is one of the major health problems in the twenty-first century due to its high incidence rate and the second leading cause of mortality globally. Approximately 15 million people die every year due to the persistence of malignant cells, and the number of cases significantly increases day by day. For these reasons, cancer diagnosis and treatment require great effort [20,21].

Surgery, chemotherapy, radiation, and hormone and targeted therapies are examples of traditional cancer treatment approaches. However, multiple short- and long-term adverse effects may significantly affect patient prognosis depending on treatment-associated clinical factors. Several natural compounds have been studied to find new therapeutic agents, among which the bioactive elements derived from plants have gained the greatest interest in the scientific community.

The scientific community currently considers chemoprevention one of the main topics in order to counteract the continuous increase of neoplasms. Natural compounds are largely diffuse due to their anticancerous ability and their potential to overcome resistance. Many scientists focused on the anticancer properties of several flavonoids. Several in vitro studies showed the activities of flavonoids on tumor cells, such as inhibition of cell growth and alteration of tumor invasive behavior [22]. Fernández et al. studied the beneficial effects of five flavonoids, together with conventional chemotherapy, on three colorectal cancer cell lines (HCT116, HT-29, and T84) [23]. Hatono’s group demonstrated the effect of isoflavones on breast cancer cell development and their impact on breast cancer treatments [24]. Goh and collaborators demonstrated the chemopreventive role of nobiletin, extracted from citrus fruit, in colon cancer cells [25].

Xhantohumol and nobiletin, are the most abundant flavonoids extracted respectively from the hop plant and red-orange. Recently, authors showed the effects of these two compounds on colon cancer cells, demonstrating that they sensitize colorectal cancer stem cells to 5-fluorouracil (5-Fu) and oxaliplatin (FOX)-based chemotherapy and affect angiogenesis [26,27].

It has been demonstrated that inflammation favors the development of cancer and metastasis. Inflammation represents the first immune response to exogenous or endogenous damage and includes the release of inflammatory cytokines and mediators such as the interleukins IL-1, IL-6, IL-10, TNF-α (Tumor Necrosis Factor α), NF-κB, NO (Nitric Oxide), iNOS (inducible Nitric Oxide Synthase), and COX (CycloOXygenase) [28]. Constitutive induction of the NF-κB pathway has been found in lung, breast, and leukemia cells, and its up-regulation correlates with tumor progression and poor prognosis [29,30].

For this reason, in the last few years, researchers’ attention has been focused on identifying natural compounds, including flavonoids, that selectively affect or inhibit the NF-κB pathway [31,32,33].

Quercetin is a 7-hydroxyflavonol isolated from numerous plant species like horse chestnut, calendula, chamomile, and *Ginkgo biloba*. Moreover, quercetin is contained in several fruits, vegetables, green tea, and in red wine [34]. It has beneficial effects on human health by mediating antioxidant activities and having a role in the modulation of metabolic pathways [35,36]. Several in vitro studies have verified the antiproliferative effects of quercetin and its effect on the expression of apoptotic genes and cell cycle-related genes. Specifically, it has been well-documented that quercetin, by modulating PI3K/AKT/NF-κB (Phosphoinositide 3-kinases/Protein kinase B/NF-κB) or STAT3 (Signal transducer and activator of transcription 3), could exert antiproliferative, anti-inflammatory, and anticancer effects and can modulate the expression of many genes [37,38].

Quercetin produces a beneficial effect through the activation of Nrf2/ARE (Nuclear factor erythroid 2-related factor 2/ARE) that promotes the expression of antioxidant enzymes like superoxide dismutase. Moreover, quercetin inhibits the expression of pro-apoptotic and pro-inflammatory genes regulated by NF-κB [39,40]. Constitutive activation of the NF-κB transcription factor was detected in acute myeloid leukemia blasts and other hematopoietic cancers as well as in various solid tumors [41]. Quercetin shows inhibitory effects on the growth of malignant cells such as ovary, liver, bladder, or colorectal cancer cells [42,43]. More recently, Rubio and colleagues showed that in vitro leukemia cells’ treatment with 25 μM of quercetin for 24 h affects leukemia cell proliferation by modulating the NF-κB/Nrf2 pathway [44]. Furthermore, quercetin, by interfering with the NF-κB signaling pathway, induces apoptosis in colon cancer cells and decreases prostate cancer cells’ survival [45,46]. More recently, Sahyon and coworkers proved in vitro that combination therapy comprising quercetin (27.98 ± 1.74 μg/mL) and sulfamethoxazole exerts anticancer effects by inducing apoptosis via caspase-3 and NF-κB gene regulation and exhibits selective toxicity against cancer cells [47]. Moreover, the authors obtained the same results in vivo by using EAC (Ehrlich ascites carcinoma)-inoculated mice as animal models. EAC is a carcinoma that can spread in the abdominal region, affecting both the liver and kidney. For their experiment, the authors treated EAC-inoculated mice with a combination of sulfamethoxazole and quercetin (200 mg/kg/day) for 14 days.

It was reported that a common dietary flavonoid, apigenin, a trihydroxyflavone extracted from plants such as parsley (*Petroselinum crispum*), celery (*Apium graveolens*), and chamomile (*Matricaria chamomilla*), may prevent cancer therapy. A recent study conducted by Shukla and coworkers revealed that in TRAMP (TRansgenic Adenocarcinoma Mouse Prostate) mouse model, treatment with apigenin (20 and 50 μg/mouse/day for 20 days) inhibits phosphorylation and degradation of IκB, leading to suppression of NF-κB activation and finally affect prostate tumorigenesis. Moreover, they observed, after apigenin treatment, a downregulation of different NF-κB-regulated genes involved in proliferation (COX-2 and cyclin D1), angiogenesis (vascular endothelial growth factor), and apoptosis (B-cell lymphoma 2, Bcl-2, and B-cell lymphoma-extra-large, Bcl-xL) [48]. Another study conducted by the same group, using human prostate cancer cell lines, demonstrated the role of apigenin cells’ treatment (2.5 to 20 μM up to 16 h) in the suppression of NF-κB signaling through direct binding and consequent inhibition of IKKa [49]. It was also found that NF-κB expression and activity decreased following apigenin treatment in colon carcinoma cells and in non-small lung cancer cells. Tong et al. showed that, in colon carcinoma cell lines, 20 μM apigenin also inhibited epithelial-mesenchymal transition by blocking NF-κB translocation to the nucleus [50]. Moreover, their in vivo experiments showed the efficacy of apigenin (300 mg/kg for two weeks) in the inhibition of the metastasis of HCT-116 cells (colorectal cancer cell line) in a xenograft model of nude mice, mediated by NF-κB signaling pathway. Recently, Chen and colleagues proved that 20 μM apigenin enhanced TRAIL-induced apoptosis by suppressing the NF-κB nuclear translocation in non-small lung cancer cells [51]. In addition, in malignant mesothelioma, apigenin treatment (50 μM for 24 h) showed anticancer effects in vitro and in vivo by inhibiting NF-κB nuclear translocation [52].

Another interesting compound is represented by Diosmetin, a monomethoxyflavone isolated from citrus fruits that are known for its anti-inflammatory and antioxidant properties [53]. Several studies proved that diosmetin has antiproliferative, antimetastasis, and pro-apoptotic effects on breast cancer and hepatocellular carcinoma cells [53,54,55]. Moreover, the cytotoxic effect of diosmetin against Colo205, HT-29, and Caco-2 cells demonstrates that it has antitumorigenesis properties against human colorectal cancer [53,56]. Koosha et al. demonstrated that diosmetin inhibits cell proliferation and activates apoptosis signaling pathways in human colorectal cancer through the inhibition of the BMP (Bone morphogenetic proteins) and NF-κB pathways [57]. They showed that in diosmetin-treated cells (3.58 µg/mL for 48 h), TNF-a receptors expression was increased, which in turn activates the extrinsic apoptosis pathway. It is well known that TNF-a is an NF-κB inducer [58,59]. In their study, despite the upregulation of TNF-a receptors, translocation of NF-κB was inhibited via overexpression of IκB-a. Moreover, the reduction in protein expression of survivin confirms NF-κB inhibition [57]. Hence, diosmetin activates apoptosis both through the engagement of apoptotic factors and inhibition of NF-κB.

Sun et al. in their study evaluated the possible anticancer effects of Baohuoside-I or Icariside II, a glycosyloxyflavone isolated from *Epimedium koreanum Nakai I*, in contrasting hepatocellular carcinoma progression [60]. They proved that the treatment with 5–10 μM for 24 h of baohuoside-I significantly inhibited the proliferation of hepatocellular carcinoma cells by inducing apoptosis and downregulating the NF-κB signaling pathway. Thus, Baohuoside-I could be a potentially novel drug, opening new possibilities in the treatment of hepatocellular carcinoma [60].

El-Shitany’s group studied the role of an interesting compound, Icariin [61]. It is a prenylated flavonol glucoside, extracted from *Epimedium koreanum Nakai*, commonly known as horny goat weed, which offers different pharmacological properties, including reduction of inflammation and antioxidant effect. This group evaluated the anti-inflammatory mechanisms of icariin, focusing on both HO-1 (heme oxygenase-1)/Nrf2 and NF-κB pathways. They evidenced that in rats with induced acute inflammation, the pre-treatment with icariin (50 mg/kg) reduced levels of several inflammatory cytokines, COX-2 gene expression, and NF-κB activation [61].

Baicalein is a trihydroxyflavone extracted from the roots of *Scutellaria baicalensis* and showed antitumor activity in many cancer types. The anticancer potential of Baicalein has been investigated in a great number of studies [62,63,64,65]. It inhibits the proliferation of human cervical cancer cells by inducing apoptosis [66]. Similarly, it was also found to be active in inhibiting human prostate and ovarian cancer cells [67,68]. In the manuscript of Sheatta and colleagues, for their in vivo experiments, EAC-inoculated mice were treated with baicalin (50 mg/kg/day for three weeks) and with baicalin and/or 5-FU. Their results proved that baicalein significantly reduced inflammation and angiogenesis via suppression of NF-κB/IL-1b and VEGF (Vascular Endothelial Growth factor) as well as apoptotic pathways activation through the up-regulation of pro-apoptotic and downregulation of antiapoptotic genes [69].

Luteolin is a tetrahydroxyflavone found in plant leaves, fruits, cloves, and foods such as chamomile tea and green pepper [70]. Several studies showed that it is involved in sensitizing cancer cells to therapeutic-induced cytotoxicity by suppressing cell survival regulated by the NF-κB pathway [10]. The study conducted by Huang and coworkers showed that treatment with 10 and 30 µM of luteolin for 24 h blocked invasion and cell cycle progression of breast cancer cell lines inhibiting the NF-κB pathway and, as a consequence, reducing c-Myc proto-oncogene and hTERT (human telomerase reverse transcriptase) expression levels [71].

Nobiletin is a natural polymethoxy flavone extracted from the fruit peel of citrus. Nobiletin is well known to show anti-inflammatory properties, and in addition, it has been revealed to suppress the proliferation of human skin, prostate, colon, and breast carcinoma cell lines [72,73]. Jiang’s group found that nobiletin was actively involved in inducing autophagy, G0/G1 cell cycle arrest, and NF-κB signaling pathway inhibition, along with reducing cell migration and invasion in human pancreatic carcinoma cells [74]. Specifically, in their experiments, the authors treated the cells with different doses of nobiletin (6.12, 12.5 and 25 μM) for 24 h, and they found a dose-dependent reduction of the invasion capability of the cells and the inhibition of the NF-κB protein expression [74]. Based on these results, nobiletin could be evaluated as a potential lead molecule for the systemic treatment of cancer and merits more investigations.

Several experimental evidence suggested that the extracts of Litchi seeds have anticancer effects and can inhibit metastatic tumor growth [75,76]. It was proved that Litchi seed extracts, particularly enriched in flavonoids, show antiprostate cancer properties both in vitro and in vivo [77]. Recently, Chang and coworkers, evaluating the effects of the treatment with 100 μg/mL of total flavonoids of litchi seeds (TFLS) for 48 h on prostate cancer cell lines, found that this compound inhibited the NF-κB signaling pathway by decreasing the phosphorylation of IκBα and NF-κB nuclear expression. Moreover, their results illustrated that NF-κB influenced EMT (epithelial-mesenchymal transition). In addition, they showed that TFLS increased the level of cleaved-PARP (Poli ADP-ribose polymerase) and Bax (Bcl-2 associated X protein) and reduced the level of Bcl-2 (B-cell lymphoma 2). Hence, their results demonstrated that TFLS exerted pro-apoptotic and antimetastatic effects in prostate cancer cells by inhibiting the activation of the AKT/NF-κB signaling pathway [78].

Recently, the possible antitumoral role of Genistein was evaluated, a 7-hydroxyisoflavone in soybeans with different biological activities [79]. It was proved that genistein inhibits carcinogenesis in animal models [80]. Research results obtained by Xie and colleagues revealed that 20 µM genistein induces multiple myeloma cell proliferation inhibition and apoptosis by suppressing the NF-κB signaling pathway [81]. More recently, the Ozturk group revealed a role of genistein in thyroid cancer cell apoptosis through the downregulation of the NF-κB pathway [82].

In recent years, it was proved the anticancer role of Wogonin, a dihydroxy- and monomethoxy-flavone extracted from *Scutellaria baicalensis Georgi*, by inhibiting NF-κB activity. In particular, results obtained by Liu and colleagues demonstrated that treatment with increasing doses of wogonin (0, 25, 50 and 100 μM, for 24 h) can act as a natural sensitizer of human chemoresistant myelogenous leukemia cells through inhibition of Nrf2 via NF-κB signaling [83].

The major polyphenolic component of dried green tea extracts is epigallocatechin-gallate (EGCG), the ester of gallic acid and epigallocatechin, which belong to the family of flavanol [84]. Several in vitro, in vivo, and clinical studies have shown multiple EGCG anticancer actions. Among them, there are antiproliferative, pro-apoptotic, antiangiogenic, and anti-invasive functions [85]. Furthermore, EGCG has been observed to impair other processes that are involved in carcinogenesis as inflammation, oxidative stress, and hypoxia, and to target tumor microenvironment components [86]. EGCG negatively modulates the expression of various transcription factors such as Sp1 (Specific protein 1), AP-1 (activator protein 1), and NF-κB, finally preventing cancer formation [87,88]. More recently, it was revealed by Tian and colleagues that 20 μM EGCG may significantly sensitize ovarian cancer cells to cisplatin through modulation of the c-Myb-induced NF-κB -STAT3 signaling pathway [89].

Anticancer properties were also demonstrated for Eupatilin and Oroxylin natural flavonoids. Wang and colleagues showed that Eupatilin, a trimethoxyflavone mainly found in *Artemisa asiatica*, could be a good anticancer compound. They proved that Eupatilin plays a role in the prevention of gastric cancer. In particular, they demonstrated that eupatilin treatment (12.5, 25 and 50 µM of for 24 h) of gastric cancer cells can reduce NF-κB activity, as well as down-regulating pro-inflammatory cytokines and metalloproteinases (MMP-2, MMP-9) [90]. Sun’s group suggests a possible role of Oroxylin A, a dihydroxy- and monomethoxy-flavone extracted from the *Oroxylum Indicum* tree and the *Scutellariae* radix, as a therapeutic candidate for the treatment of breast cancer. Their data reported that 24 h treatments with 20 μM of Oroxylin A can inhibit the invasion, cell proliferation, migration, and EMT by inactivating the NF-κB signaling pathway in human breast cancer cells [91].

Recently, another natural flavonoid, the astragalin, a trihydroxyflavone extracted from different medicinal plants such as *Eucommia ulmoides*, *Moringa oleifera*, *Morus alba*, and *Radix astragali*, has been reported to induce cell death in a caspase-dependent way enhancing the sensibility of lung cancer cells to TNF-a and inhibiting NF-κB activation [92]. Specifically, Chen and coworkers showed, by treating lung cancer cells for 24 h with different astragalin doses (2.5, 5, 10, 20 μg/mL), that this compound inhibits NF-κB/p65 nuclear translocation and IκBa degradation in a dose-dependent manner.

**Figure 2:**
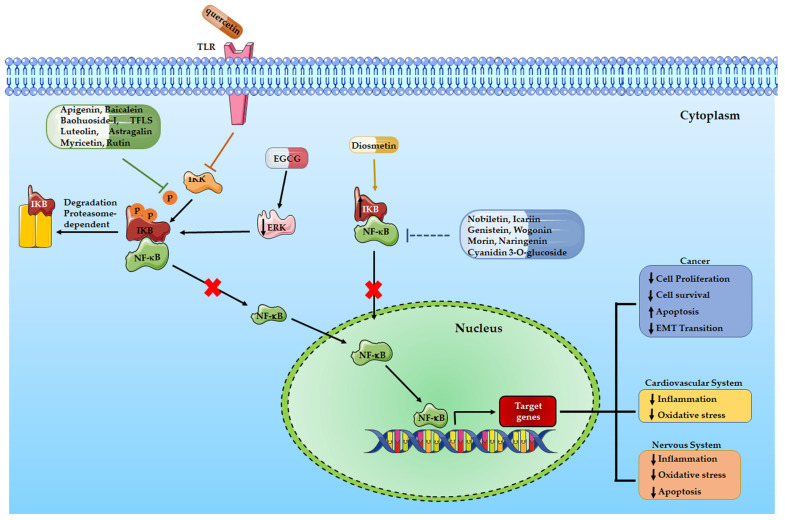
A schematic illustration of the effects of flavonoids in different human diseases mediated by NF-κB signaling pathway.

The literature data cited in this paragraph and summarized in Table 1 let us suppose that natural compounds widely present in the daily human diet, such as flavones and flavonols, through modulation of the NF-κB signaling pathway, could play a critical role in inhibiting tumor growth and could be considered as natural chemopreventive compounds (Figure 2).

## 4. Flavonoids and Cardiovascular Diseases: Role of NF-κB Signaling Pathway

Nowadays, cardiovascular disease (CVD) is the leading cause of death, especially in Europe, where it remains the primary cause of 42% of mortalities in men and 52% in women [93]. In the United States, it is estimated that 31% of all global deaths in 2016 were from CVDs, mainly heart attacks and stroke [94].

Inflammation is a key risk factor for CVD. A good deal of evidence demonstrates that chronic inflammation can affect atherosclerosis, myocardial infarction, and heart failure [95]. The upregulation of cytokines in the heart activates intracellular signaling pathways such as NF-κB, thus finally altering cardiac cell biology. It is well known that NF-κB activity is strictly correlated with the development and progression of inflammation and cardiovascular damage, as discussed by Fiordelisi and collaborators in their recent review [96].

Atherosclerosis (AS) is the primary cause of CVD that can determine myocardial infarction. AS can affect immune inflammatory activity through NF-κB activity, thus finally contributing to platelet migration and aggregation [97].

Platelet activation can induce aggregation and thrombus formation, ultimately contributing to ischemia and myocardial infarction. Several drugs are available to treat and prevent platelet hyperactivation, but they have many collateral negative effects. Diet and lifestyle have an impressive effect on LDL (Low-density lipoprotein)-cholesterol levels and CVD risk. For these reasons, recent studies reported the cardioprotective effects of medicinal plants and the Mediterranean diet in primary and secondary CVD prevention [98]. Hypertension is one of the most common diseases and a major risk factor for stroke and myocardial infarction; many patients affected by cardiovascular diseases are characterized by hypertension and/or cardiac dysfunction. In particular, hypertension can lead to a sustained increase in heart pressure overload, resulting in excessive proliferation and accumulation of collagen fibers in the interstitium and perivascular vessels, which can lead to myocardial fibrosis. For this reason, it is very important to decrease myocardial fibrosis, thus reducing the risk of cardiovascular disease.

Several epidemiological studies demonstrated the benefits of a diet enriched with flavonoids, especially for heart diseases.

The effect of Quercetin on CVD is well known; in fact, quercetin can reduce the expression of adhesion molecules and inflammatory markers [99]. It has been demonstrated that 25 μM of quercetin modulates TLR-NF-κB (Toll-Like Receptor-Nuclear Factor kappa B) signaling pathway on endothelial cells in vitro, thus affecting the expression of adhesion molecules involved in the leukocyte-endothelial interaction. Moreover, the authors demonstrated the anti-inflammatory activity of quercetin in rats treated for 60 days with 25 mg quercetin/kg body weight in a condition of a hypercholesterolemic diet [100]. Furthermore, by reducing MMP2 (Matrix Metalloproteinase 2) activity and oxidative stress, chronic treatment with quercetin (10 mg/kg/day) ameliorates hypertension-induced coronary hypertrophic remodeling in renovascular hypertensive rats, even if it does not reduce cardiac dysfunction [101]. An interesting study on patients with stable coronary artery disease demonstrated that quercetin therapy reduces the levels of IL-1b in the blood but also the level of IκBa mRNA, thus affecting the transcriptional activity of NF-κB signaling and finally ameliorating the indicators of chronic systemic inflammation after two-months of quercetin treatment at the daily dose of 120 mg [102].

Some studies suggested the involvement of the TGF-b (tumor growth factor b)/Smad and the NF-κB signaling pathways in the progression of myocardial fibrosis as well as the role of pro-inflammatory cytokines in the development and progression of myocardial fibrosis [95,96]. Therefore, as discussed before, an alternative strategy for the treatment of CVD, such as myocardial fibrosis, is the inhibition of inflammation.

Icariside II, the main metabolite of Icariin, is native to Korea and China, where it is well known as a nutraceutical compound in functional foods or as a phytopharmaceutical agent with anti-inflammatory and antioxidant activities that protects against cardiovascular diseases [103,104]. It is demonstrated that Icariin inhibits IL1β-induced activation of NF-κB-related signaling pathways, thus playing a significant anti-inflammatory role [105]. Fe Shu and collaborators demonstrated that Icariside II chronic treatment (4, 8, and 16 mg/kg) reduces myocardial fibrosis in spontaneously hypertensive rats through suppression of NF-κB signaling and the TGF-b1/Smad2 signaling pathways [106].

One of the major negative effects of Doxorubicin (DOXO) treatment in anticancer therapy is cardiotoxicity which can occur after 3 days (acute cardiotoxicity) or after 30 days of treatment (chronic cardiotoxicity) [107]. Doxorubicin is firstly responsible for inflammatory effects in the myocardium, characterized by the increased expression of NF-κB; moreover, cardiac inflammation contributes to cardiotoxicity. Proanthocyanidins are a class of oligomeric flavonoids derived from catechins and epicatechins, found in many plants, such as cranberry, blueberry, and grape seeds. Proanthocyanidins derived from grape seed extracts exert antioxidant effects, may reduce neutrophil infiltration and cyclooxygenase expression and, finally, reduce the adverse effects of doxorubicin treatment in anticancer therapy [108]. It was recently demonstrated that the NF-κB pathway is responsible for the prophylactic effects of proanthocyanidin extract against DOXO-induced heart injury and fibrosis [109]. Rats intraperitoneally injected with DOXO (10 mg/kg on Days 3, 9, 15, and 21 of the experiment) and proanthocyanidin extract (50 mg/kg/once daily) for 4 weeks showed a significant reduction of DOXO-induced cardiomyocyte inflammation, thus suggesting a possible role of proanthocyanidin extract against DOXO-induced cardiac toxicity [109].

Morin, a pentahydroxyflavone isolated from Chinese herbs of the *Moraceae* family and also present in many fruits and wines, can exert similar effects. Kuzu and collaborators demonstrated that Morin significantly decreases the expression of NF-κB, TNF-a, and IL-1β in DOXO-treated rats, inhibiting DOXO-induced oxidative stress and heart damage [110]. For the experiments reported in the manuscript, rats were treated with 50–100 mg/kg of morin (orally administered) for 10 days and with DOXO 40 mg/kg body weight by single dose intraperitoneal injection on the 8th day of the study. After analysis of different inflammation and cardiac markers,, the authors demonstrated the protective role of morin against DOXO-induced heart damage [110].

Curcumin, extracted from the rhizomes of *Curcuma longa,* which belongs to the ginger family, is well known as the main component of the dietary spice turmeric. Several studies demonstrated its antioxidant, anti-inflammatory and anticancer effects through inhibition of the NF-κB pathway [111,112,113]. Benzer et al. obtained similar results demonstrating the beneficial effect of curcumin against DOXO-induced cardiotoxicity in rats [114]. For their experiments, rats were treated orally for 7 days with 100–200 mg/kg body weight of curcumin, and the authors induced cardiotoxicity by injection of the single dose of 40 mg/kg of DOXO on the 5th day. Rats were sacrificed at day, and several analyses demonstrated the cardioprotective effects of curcumin after DOXO treatment [114].

Finally, we can conclude that flavonoids, as well as many other natural compounds, are able to decrease the risk of several CVD by reducing the activation of inflammatory pathways and especially of the NF-κB signaling pathway (Table 2 and Figure 2).

## 5. Flavonoids and Neurodegenerative Diseases: Role of NF-κB Signaling Pathway

Neuroinflammation is one of the main causes of different neurodegenerative diseases such as dementia, atherosclerosis, and Alzheimer’s disease (AD). Several signals can interfere with the physiological communication between neurons and glial cells. NF-κB activation is mediated by Toll-like receptors (TLRs) that mediate the intracellular inflammatory response. Focusing on the brain, TLRs are mainly expressed in microglia and astrocytes. Once activated, the microglia can induce the expression of the NF-κB pathway and the subsequent synthesis of pro-inflammatory mediators. These pro-inflammatory cytokines finally activate kinases responsible for the hyperphosphorylation of proteins, such as tau protein, involved in the stability of cytotypes of the central nervous system. The tau proteins, in fact, become insoluble, aggregate, and form the neurofibrillary tangles that destroy the functionality of the cells. Dementia, AD, or Parkinson’s disease are specifically correlated with tau hyperphosphorylation.

Several studies described the role of NF-κB signaling in neurodegenerative diseases [115]. For example, in the brain section of Parkinson’s patients, it is demonstrated a huge increase in NF-κB activation and a strong nuclear immunoreactivity of p65 [116]. Recently, Debashis Dutta et al. demonstrated that, by specifically targeting the TLR2/NF-κB pathway, it is possible to reduce the a-synuclein spreading in vitro and in vivo, finally contributing to the treatment of neurodegenerative diseases such as Parkinson’s disease (PD) [117]. Clinical studies highlight the preventive effects of correct and healthy nutrition in the field of neurodegenerative diseases and especially AD or atherosclerosis [118,119]. Many nutraceutical bioactive principles have antiplatelet activity as well as anti-inflammatory activities [118].

It has recently been demonstrated that several flavonoids and tea catechins have a protective role toward neurons and tau protein hyperphosphorylation, mainly inhibiting the activity of MAPK (Mitogen-Activated Protein Kinase) and GSK3 pathways (Glycogen Synthase Kinase-3) [120,121,122,123]. Hung and collaborators demonstrated the antioxidant effects of quercetin. Specifically, quercetin treatment for 24 h (2.5–10 uM) and subsequent exposure to oxidized LDL protects endothelial cells from damage induced by oxidized LDL. Interestingly, quercetin exerts these effects through the activation of Sirtuin1 (SIRT1), which is involved in the regulation of several chronic conditions, such as neurodegenerative diseases [124].

Naringenin is a flavanone found in different herbs and fruits, such as grapefruit, bergamot, tart cherries, and tomatoes. It is well known that naringenin has antibacterial activity but can also reduce hepatitis C virus production in liver cells in vitro [125,126]. Importantly, Naringenin represents a promising compound in the treatment of AD because it activates the expression of Amiloid-b degradation enzymes and induces M2 polarization of microglia [127]. Sarubbo and collaborators demonstrated that naringenin, as well as quercetin, negatively regulates inflammatory pathways, thus decreasing TNF-a, IL-6, and IL-1 expression through the regulation of the nuclear translocation of p65 and the increased expression of SIRT1 in the hippocampus of rat [128]. As discussed previously, SIRT1 has a neuroprotective function and regulates NF-κB expression, while aging reduces SIRT1 hippocampal expression. Old rats were subjected to chronic treatment with naringenin or quercetin (20 mg/kg/day) for 28 days. This chronic treatment, due to the antioxidant properties of polyphenolic compounds, stabilize hippocampal SIRT1 expression, thus affecting NF-κB signaling and the inflammation pathway [128].

Epigallocatechin is the most abundant component of tea catechins, found in solid green tea extract. For this reason, it is responsible for green tea’s antioxidant, anti-inflammatory, and antiapoptotic activities [129]. Epigallocatechin 3-gallate (EGCG) overpasses the blood–brain barrier and reduces the β-amyloid (Aβ) accumulation through the suppression of the ERK/NF-κB pathway, finally exerting a neuroprotective effect [130,131].

Chang and Rong demonstrated that EGCG inhibited neurotoxic Aβ accumulation in a model of a senescent mouse (SAMP8, Senescence-accelerated mouse prone 8) subjected to EGCG chronic treatment of 5 and 15 mg/kg for 60 days. Moreover, the authors demonstrated that EGCG induced the expression of neprilysin, a protease involved in brain Aβ metabolism in vivo, thus reducing the Aβ accumulation [132].

Myricetin is an hexahydroxyflavone present in several vegetables and fruits [133]. The compound exerts various beneficial effects such as antihypertensive, anti-inflammatory, and immunomodulatory [134,135]. As discussed, neuroinflammation is caused by microglia activation. Long Sun et al. utilized a rat model of permanent middle cerebral artery occlusion (pMCAO) and administered three different concentrations of myricetin (1–5–25 mg/kg) once per day for 1 week. They demonstrated that myricetin reduces ischemic cerebral injury in the rat middle cerebral artery occlusion model by decreasing the expression of several pro-inflammatory cytokines, such as IL-1b, IL-6, TNF-a, the phosphorylation of p38 MAPK, and the level of NF-κB/p65 [136]. Jang and collaborators have recently demonstrated the inhibitory effects of Myricetin on microglia neuroinflammation through the production of pro-inflammatory molecules and cytokines [137]. The authors demonstrated their results in vitro on microglial BV-2 cells treated with myricetin 10–25 μM for 1 h and then stimulated with LPS. Moreover, myricetin (50–100 mg/kg), once a day for 7 days, inhibited LPS-induced macrophages and microglial activation in the hippocampus and cortex of mice in vivo [137]. Kaewmool et al. obtained similar results studying the effects of Cyanidin 3-O-glucoside, anthocyanins responsible for the red and blue pigments of different fruits and well known for its neuroprotective effects [138]. Authors demonstrated that cyanidin treatment (2.5–5–10 μM) for 4 h inhibits the NF-κB and p38 pathways, thus affecting the expression of pro-inflammatory cytokines in microglial BV2 cells [138,139].

Recently, Chiocchio et al. demonstrated the neuroprotective role of a flavonoid blend contained in leaves of *Castanea sativa* and mainly enriched in astragalin, isorhamnetin glucoside, and myricetin. Authors demonstrated that chestnut extracts significantly decrease the mRNA expression of pro-inflammatory cytokines and the activation of the NF-κB pathway on BV2 microglial cells treated for 3 h with 0.5 mg/mL of the extract [140].

It is well known that PD patients have a high level of pro-inflammatory cytokines in their brains and that bacterial LPS (lipopolysaccharides) can induce microglia activation, inflammatory cytokines expression, and PD-like symptoms in animal models [141]. Hartmann and colleagues demonstrated that activation and nuclear translocation of NF-κB are specifically involved in this process [142]. Patel and Singh recently demonstrated that apigenin treatment (25–50 mg/kg) for 2 weeks attenuates LPS-induced parkinsonism in experimental rats targeting TLR/NF-κB and Nrf2/HO-1 signaling pathways. Apigenin reduces neuroinflammation in rat striatum as demonstrated by a significant decrease in TNF-a, IL-1b and IL-6 expression. Moreover, it reduces the nuclear translocation of phospho-NF-κB in the rat brain, finally exerting a neuroprotective effect [143].

Rutin, a natural flavonoid glycoside derived from quercetin, exerts similar neuroprotective effects on different neurodiseases, including AD and PD [144]. Sun and collaborators demonstrated that rutin treatment (100 mg/kg) for 30 days prevents neuroinflammation in mouse models of AD by decreasing IL-1b and TNF-a production. Interestingly, authors evaluated the level of IKK-b, p65, and phospho-p65 in the brain protein lysates of mice to demonstrate that the rutin’s neuroprotective effects are mediated by the downregulation of NF-κB activation [145].

In 2014, Wang and collaborators showed that Genistein, at the dose of 10 mg/kg once daily for 2 weeks, ameliorated brain damage [146]. Recently it has been demonstrated that genistein reduces the inflammation of the Hippocampus in cecal ligation and puncture (CLP)-induced septic rats treated with a dose of 15 mg/kg daily for 10 days. In this study, authors focused on the hippocampus NF-κB activation showing that genistein reduces the degradation of IκBa and the nuclear translocation of NF-κB [147].

It is known that nobiletin showed antidementia properties attenuating cognitive impairment in the AD mice model [148,149]. Qi et al. recently demonstrated that nobiletin treatment (25–50–100 μM) for 4 h counteracts the microglia activation and the secretion of pro-inflammatory mediators in BV2 microglia cells. Moreover, the authors demonstrated the protective effects of nobiletin on mice treated with a dose of 100 mg/kg once daily for 7 weeks. These effects are mediated by the modulation of MAPKs, PI3K/AKT, and NF-κB signaling pathways [150]. Ghasemi-Tarie showed similar results in the AD rat model where nobiletin treatment (mg/kg/day) for 7 days prevents amyloid-induced cognitive defects by blocking p65 nuclear translocation and IκBa phosphorylation [151].

Here, we summarize the role of some natural compounds that, through affecting inflammatory pathways, and specifically the NF-κB pathway, can reduce neuroinflammation and subsequent neuronal degeneration (Table 3 and Figure 2).

## 6. Methods

The literature review was conducted using recommended five-step scoping review guidelines [153]: (1) identify a research question (Roles of NF-κB signaling pathways in mediating the effects of flavonoids on human diseases); (2) identify relevant studies; (3) select relevant studies; (4) chart data from these studies; and (5) collate, summarize, and report the results.

We have identified studies from the PubMed/Medline database about flavonoids and NF-κB signaling pathways in human diseases. The aim was to access original research on subjects related to human pathology. Keywords used to search the database were Flavonoids, NF-κB, cancer, neurological diseases, and cardiovascular diseases. We have excluded non-English reviews, and we included papers published within recent 5 years (except for the cardinal papers on the topic).

## 7. Conclusions

To date, NF-κB represents a key signaling molecule that regulates the expression of a great number of genes, thus modulating several cell signaling pathways such as cell survival and proliferation pathways or immune regulatory and inflammation pathways. For this reason, in the last decades, researchers’ attention was focused on the possibility that NF-κB could be a good target for several diseases. Therefore, one of the researcher’s challenges is the development of NF-κB inhibitors to treat different diseases and avoid the risk of side effects related to conventional therapy. A healthy diet is recognized all over the world to prevent or ameliorate several diseases, such as cardiovascular and neurodegenerative diseases, or cancer and its progression. Several natural compounds are now being explored for their capability to affect and reduce the progression of many diseases with low collateral effects or toxicity. Moreover, recent manuscripts describe the role of flavonoids as negative regulators of the NF-κB signaling pathway. For these reasons, the scientific community is now focusing on flavonoids contained in many plants, fruits, and beverages as a possible alternative strategy to overcome or prevent different pathologies. The literature data reported in this review suggest the potential capability of flavonoids to modulate pathological diseases such as cancer, cardiovascular and neurodegenerative diseases by affecting directly or indirectly the NF-κB pathway (Figure 2). Furthermore, the researcher’s results might provide substantial support for further investigation to assess the efficacy and safety of flavonoid compounds as part of a healthy diet or as co-treatment of human cancers and cardiovascular and neurodegenerative diseases.

## 8. Limitations and Future Perspectives

It is well known that flavonoids are lipophilic compounds, but their interaction with biological membranes is not clear and needs to be better clarified. Many flavonoids interact with receptors on the membrane, protein components of the membrane, and often with lipid rafts. The interaction with the membrane and/or the speed of the diffusion depends on the different compositions of the membrane in each tissue and, on the other hand, on the specific chemical structure of each flavonoid [154]. The presence of definite groups facilitates the adsorption of the compounds, e.g., gallate groups in catechins make EGCG better adsorbed by the membrane [155,156,157]. Flavonols, because of their planar structure, interact with the membrane with higher affinity than flavanones, even if flavanones are more hydrophobic [158].

Another limitation, that we have to take into account if we want to consider flavonoids as food supplements, is the toxicity of these compounds. Even if the beneficial properties of all these flavonoids are clear, it is important to consider that they have to pass clinical trials for efficacy and toxicity in humans. Many flavonoids have been demonstrated to be mutagenic in mammalian cells or in mice [159,160,161]. Recently Zhang and colleagues analyzed the toxicities of four flavonoids (luteolin, apigenin, quercetin, and genistein), demonstrating, through in silico, in vitro, and in vivo analysis, the developmental and mutagenic toxicity together with estrogen activity that makes them dangerous, especially in young people [161].

Further investigations to optimize the bioavailability, biodistribution, and safety of the compounds for future human studies are needed. Specifically, it is necessary: (i) to clarify how each flavonoid, according to its specific chemical structure, can cross the membrane; (ii) that the scientific community deepens knowledge and clarifies the real toxicity of each compound.

## Figures and Tables

**Table 1:** Summary of the effects of flavonoids on cancer mediated by the NF-κB signaling pathway.

Flavonoids	Subgroups	Chemical Structure	Activity as NF-κB Signaling Inhibitor with Proven Anticancer Effects	Refs.
Quercetin	Flavonol	C_15_H_10_O_7_	Leukemia cells. Cervical, non-small lung, colon, liver, and prostate cancer cells	[42,43]
Apigenin	Flavone	C_15_H_10_O_5_	Prostate adenocarcinoma, colon, prostate, and lung cancer cells. Mesothelioma.	[56,57,58,59]
Diosmetin	Flavone	C_16_H_12_O_6_	Colorectal cancer cells	[53,54,55,57]
Baohuoside-I	Flavonol	C_27_H_30_O_10_	Hepatocellular carcinoma,	[60]
Baicalein	Flavone	C_15_H_10_O_5_	Breast cancer	[69]
Luteolin	Flavone	C_15_H_10_O_6_	Glioblastoma cells, breast and pancreatic cancer cells, hepatocarcinoma cells	[10,71]
Nobiletin	Flavone	C_21_H_22_O_8_	Pancreatic carcinoma, and colorectal cancer cells	[74]
TFLS	Flavone	C_15_H_10_O_5_	Prostate cancer cells	[78]
Genistein	Flavone	C_15_H_10_O_5_	Breast, thyroid, and colon cancer cells and multiple myeloma cells	[81,82]
Wogonin	Flavone	C_16_H_12_O_5_	Hepatocellular carcinoma, colon cancer, and myelogenous leukemia	[83]
EGCG	Flavan-3-ol	C_22_H_18_O_11_	Ovarian cancer cells	[89]
Eupatilin	Flavone	C_18_H_16_O_7_	Gastric cancer cells	[90]
Oroxylin A	Flavone	C_16_H_12_O_5_	Breast cancer cells	[91]
Astragalin	Flavone	C_21_H_20_O_11_	Colon and lung cancer cells	[92]

**Table 2:** Summary of the effects of flavonoids on cardiovascular diseases mediated by the NF-κB signaling pathway.

Flavonoids	Subgroups	Chemical Structure	Effects on NF-κB Signaling with Proven Positive Effect on Cardiovascular Diseases	Refs.
Quercetin	Flavonol	C_15_H_10_O_7_	Hypertension, coronary artery diseases	[101,102]
Icariside II	Flavonol	C_27_H_30_O_10_	Myocardial fibrosis	[106]
Proanthocyanidin	polyphenols		Heart injury and fibrosis	[109]
Morin	Flavone	C_15_H_10_O_7_	Heart damage	[110]
Curcumin	Diarylheptanoid		Cardiotoxicity	[111]

**Table 3:** Summary of the effects of flavonoids on neurodegenerative diseases mediated by the NF-κB signaling pathway.

Flavonoids	Subgroups	Chemical Structure	Effects on NF-κB Signaling with Proven Positive Effect on Neurodegenerative Diseases	Refs.
Quercetin	Flavonol	C_15_H_10_O_7_	Atherosclerosis	[132]
Apigenin	Flavone	C_15_H_10_O_5_	Parkinson disease	[48,49,50,51,143]
Naringenin	Flavanones	C_15_H_12_O_5_	Alzheimer disease cognitive impairment	[127,152]
EGCG	Flavan-3-ols	C_22_H_18_O_11_	Alzheimer disease	[138]
Myricetin	Flavonols	C_15_H_10_O8	Ischemic cerebral injury, microglia inflammation	[136,137]
Cyanidin 3-O-glucoside	Anthocyanin	C_21_H_21_ClO_11_	microglia inflammation	[138,139]
Rutin	Flavonols	C_27_H_30_O_16_	Alzheimer disease	[145]
Genistein	Flavone	C_15_H_10_O_5_	Hippocampus inflammation	[147].
Nobiletin	Flavone	C_21_H_22_O_8_	Microglia inflammation Alzheimer disease	[150,151]

## Data Availability

No new data were created or analyzed in this study. Data sharing does not apply to this review article.

## Abbreviations

NF-κB	Nuclear Factor-κB
USDA	United States Department Of Agriculture
UV	Ultraviolet
IKK	IκB Kinase
IκB	Inhibitor of NF-κB
NLS	Nuclear localization signal
TNFR	Tumor Necrosis Factor Receptor
NIK	NF-κB-inducing kinase
5-FU	5-Fluorouracil
TNF-α	Tumor Necrosis Factor α
IL-1	Interleukin-1
IL-6	Interleukin-6
IL-10	Interleukin-10
NO	Nitric Oxide
iNOS	Inducible Nitric Oxide Synthase
COX	CycloOXygenase
PI3K	Phosphoinositide 3-kinases
AKT	Protein kinase B
STAT3	Signal transducer and activator of transcription 3
Nrf2	Nuclear factor erythroid 2-related factor 2
ARE	Antioxidant response element
EAC	Ehrlich ascites carcinoma
TRAMP	TRansgenic Adenocarcinoma Mouse Prostate
Bcl-2	B-cell lymphoma 2
Bcl-xL	B-cell lymphoma-extra-large
TRAIL	Tumor-necrosis factor-related apoptosis-inducing ligand, CD253
BMP	Bone morphogenetic proteins
HO-1	Heme oxygenase-1
VEGF	Vascular Endothelial Growth factor
hTERT	Human telomerase reverse transcriptase
TFLS	Total flavonoids of litchi seeds
EMT	Epithelial-mesenchymal transition
PARP	Poli ADP-ribose polymerase
Bax	Bcl-2 associated X protein
EGCG	Epigallocatechin-gallate
Sp1	Specific protein 1
AP-1	Activator protein 1
MMP-2	Metalloproteinase-2
MMP-9	Metalloproteinase-9
CVD	Cardiovascular disease
AS	Atherosclerosis
LDL	Low-density lipoprotein
TLR	Toll-Like Receptor
TGF-β	Tumor growth factor-β
DOXO	Doxorubicin
AD	Alzheimer’s disease
PD	Parkinson’s disease
MAPK	Mitogen-Activated Protein Kinase
GSK3	Glycogen Synthase Kinase-3
SIRT1	Sirtuin1
Aβ	β-amyloid
Erk	Extracellular signal-regulated kinases
SAMP8	Senescence-accelerated mouse prone 8
pMCAO	Permanent middle cerebral artery occlusion
LPS	Lipopolysaccharide
CLP	Cecal ligation and puncture
